# Preoperative Low Serum Basophil Levels Predict Poor Prognosis for the Patients with Esophageal Squamous Cell Carcinoma

**DOI:** 10.5761/atcs.oa.26-00001

**Published:** 2026-03-04

**Authors:** Fumiaki Shiratori, Satoshi Yajima, Takashi Suzuki, Yoko Oshima, Teruki Yamakawa, Yuichiro Ohtsuka, Hideaki Shimada

**Affiliations:** 1 Department of Surgery, School of Medicine, Toho University, Tokyo, Japan; 2 Department of Health Management Center, Funabashi Central Hospital, Funabashi, Chiba, Japan

**Keywords:** basophil, esophageal cancer, diagnosis, prognosis, squamous cell carcinoma

## Abstract

**Purpose:**

A decrease in peripheral basophil count has recently been suggested as a potential marker of poor prognosis in malignancies. This study aimed to determine the optimal cutoff value for basophil count and assess its clinicopathological and prognostic significance in esophageal squamous cell carcinoma (ESCC).

**Methods:**

We retrospectively analyzed 194 patients with ESCC (157 men, 37 women; mean age, 67 years [range, 34–88]) who underwent curative surgery between 2010 and 2020, including 100 who received neoadjuvant chemotherapy. Receiver-operating characteristic curve analysis identified an optimal basophil cutoff value of 17.4/μL. Patients were divided into low- and high-basophil groups, and clinicopathological factors and prognosis were assessed using univariate and multivariate analyses.

**Results:**

Low-basophil counts were significantly correlated with low neutrophil counts but not with C-reactive protein level. Multivariate analysis to predict overall survival identified deep invasion, elevated C-reactive protein, and low-basophil count as independent predictors of a poor prognosis (*P* <0.05).

**Conclusion:**

Low preoperative basophil count is an independent adverse prognostic factor in ESCC. As basophil count was not correlated with C-reactive protein, it may provide prognostic value beyond conventional inflammation-based markers and could represent a simple, low-cost biomarker to aid risk stratification in the preoperative setting.

## Abbreviations

ESCC
: esophageal squamous cell carcinoma
CRP
: C-reactive protein
NAC
: neoadjuvant chemotherapy
ROC
: receiver-operating characteristic
OS
: overall survival
NLR
: neutrophil-to-lymphocyte ratio
PLR
: platelet-to-lymphocyte ratio

## Introduction

In patients with solid tumors, various blood biochemical parameters have been associated with prognosis.^1,2)^ The neutrophil-to-lymphocyte ratio (NLR), platelet-to-lymphocyte ratio (PLR), serum albumin, lymphocyte-to-monocyte ratio, and C-reactive protein (CRP) are among the most widely investigated markers.^3–8)^ By contrast, the clinical relevance of basophils remains largely unexplored. Basophils, the least abundant circulating leukocytes (<1%), have traditionally been considered as effector cells in immediate hypersensitivity reactions.^9)^ Moreover, attention has recently been drawn to their potential role in tumor immunity.

Basophils are believed to induce T-helper type 2–type immune responses by producing cytokines such as interleukin-4 (IL-4) and IL-13, which may indirectly modulate the activation of dendritic cells and CD8^+^ T cells.^10)^ Some studies have further suggested that intratumoral basophil infiltration can suppress T-cell recruitment and impair tumor rejection.^11,12)^ Although intratumoral infiltration of basophils might lead to a decrease in peripheral basophil count, a consensus has not yet been reached.

Unlike conventional inflammation-based biomarkers, basophils may represent a novel prognostic marker of tumor immunity. Several studies have examined their clinical relevance in gastrointestinal cancers, including esophageal, gastric, and colorectal cancer, but their precise role remains unclear.^13–17)^ We previously demonstrated that a low preoperative basophil count was associated with poor prognosis in gastric cancer.^15)^ To date, only one study by Maruyama et al. has addressed the role of basophils in esophageal squamous cell carcinoma (ESCC), reporting that patients with counts below 22/mm^3^ had significantly worse outcomes.^13)^ However, the optimal cutoff value for basophil count has not been established. Furthermore, prior studies on basophil count in esophageal cancer did not stratify analyses according to treatment strategy, such as preoperative chemotherapy or upfront surgery.^13)^

Therefore, this study aimed to identify a prognostically meaningful cutoff value for peripheral basophil count in patients with ESCC undergoing surgical treatment. Additionally, subgroup analyses were performed to evaluate overall survival (OS) separately in patients who received neoadjuvant chemotherapy (NAC) and those who underwent upfront surgery.

## Materials and Methods

**Fig. 1:**
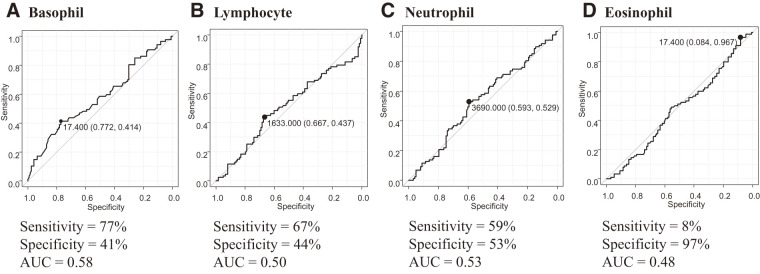
The AUC to determine the cutoff value of (**A**) basophil, (**B**) lymphocyte, (**C**) neutrophil, and (**D**) eosinophil counts to predict overall survival. AUC: area under the receiver-operating characteristic curve

The optimal cutoff value for basophil count was determined to be 17.4 cells/µL using receiver-operating characteristic (ROC) curve analysis (**Fig. 1**). Similarly, cutoff values were calculated for other leukocyte subsets: 1633 cells/μL for lymphocytes, 3890 cells/μL for neutrophils, and 17.4 cells/μL for eosinophils, all derived from ROC curve analysis. The optimal cutoff value for CRP level was set at 0.2 mg/dL, the institutional standard at our hospital.

### Patients

**Fig. 2:**
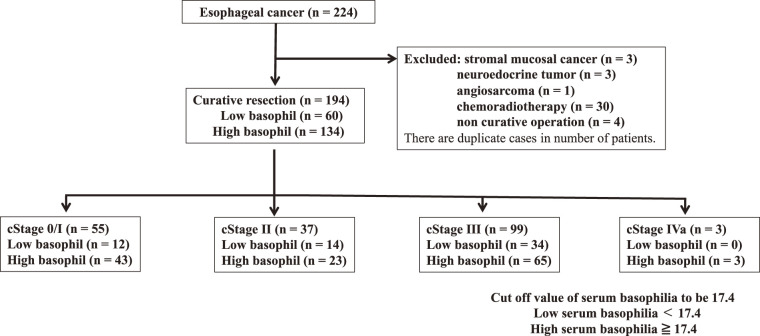
A flowchart showing patient selection for this study.

A total of 194 patients (157 men and 37 women) who underwent curative esophagectomy at Toho University Omori Medical Center (Tokyo, Japan) between January 2010 and December 2020 were analyzed. The median age was 67 years (range, 34–88). Eligible patients had histologically confirmed primary ESCC. Patients with active synchronous or metachronous malignancies within a 5-year disease-free interval were excluded to minimize confounding effects on OS (**Fig. 2**). We confirmed that patients with bronchial asthma, allergic rhinitis, atopic dermatitis, food or drug allergies requiring ongoing treatment, autoimmune diseases, chronic inflammatory diseases, active or chronic infections (including tuberculosis, chronic hepatitis, and human immunodeficiency virus infection), and a history of acute infections were not included. Of the 194 patients, 100 received NAC. The regimens included CF (cisplatin 80 mg/m² on day 1 plus 5-fluorouracil 800 mg/m² on days 1–5 every 3 weeks). Preoperative clinical staging, classified according to the 11th edition of the Japanese Classification of Esophageal Cancer, was pathological stage 0/I in 55 patients, stage II in 37 patients, stage III in 99 patients, and stage IV in 3 patients. Tumor resectability and extent of local or distant progression were assessed using computed tomography. Tumors with distant metastasis were considered unresectable. All patients underwent esophagectomy with radical lymphadenectomy. The median follow-up period was 44 months.

In this study, serum basophil counts were assessed preoperatively. For patients who underwent neoadjuvant therapy, basophil counts were measured prior to treatment initiation. The mean preoperative basophil count was 25.5 ± 28.2 cells/μL (range, 2.9–177.5). Patients were divided into 2 groups using the ROC-derived cutoff of 17.4 cells/μL: a high-basophil group (≥17.4) and a low-basophil group (<17.4). OS and clinicopathological characteristics were then compared between these groups. The cutoff value for CRP was set at 0.2 mg/dL, in accordance with the assay manufacturer’s instructions.

### Statistical analysis

Fisher’s exact test was used to compare categorical variables. OS rates were estimated using the Kaplan–Meier method, and survival curves were compared with the log-rank test. Multivariate analysis was performed using a Cox regression model to identify prognostic factors associated with OS. Two-tailed *P*-values <0.05 were considered indicative of statistical significance. The optimal cutoff value for basophil count in relation to OS was determined by ROC curve analysis. All statistical analyses were performed using EZR (Saitama Medical Center, Jichi Medical University, Saitama, Japan),^18)^ a graphical user interface for R (version 2.13.0; The R Foundation for Statistical Computing, Vienna, Austria). EZR is a modified version of R Commander (version 1.6–3) designed to facilitate biostatistical analyses. Differences with *P*-values <0.05 were considered statistically significant.

## Results

### Comparison of clinicopathological characteristics between low- and high-basophil count groups

**Fig. 3:**
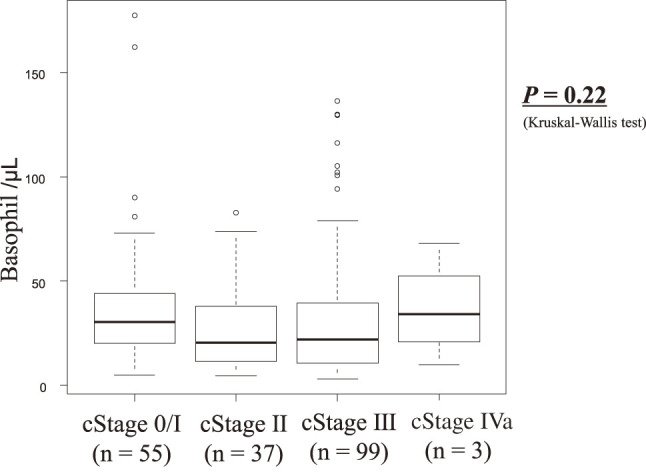
Basophil count according to the tumor stages.

The clinicopathological characteristics of the low- and high-basophil count groups are compared in **Table 1**. No significant associations were found with any clinicopathological factors. The TNM (Tumor, Node, Metastasis) stage showed no association with basophil count (**Fig. 3**).

**Table 1:** Comparison of clinicopathological characteristics between low- and high- basophil groups

Variables	Number of patients	Low-basophil group (n = 60)	High-basophil group (n = 134)	*P*-value^a^
Age (years old)				
<65	76	24	52	0.88
≥65	118	36	82	
Gender				
Female	37	9	28	0.43
Male	157	51	106	
Tumor depth				
cT0cT1	56	15	41	0.50
cT2cT3cT4	138	45	93	
Nodal status				
cN0	76	20	56	0.34
cN1cN2cN3	118	36	82	
C-reactive protein (mg/dL)				
<0.2	108	35	73	0.64
≥0.2	86	25	61	
Neutrophil (/μL)				
<3690	106	42	64	<0.01
≥3690	88	18	70	
Lymphocyte (/μL)				
<1633	115	42	73	0.06
≥1633	79	18	61	
Eosinophil (/μL)				
<17.4	12	4	8	1
≥17.4	182	56	126	

^a^Fisher’s exact probability test.

### Effects of low-basophil count on OS

**Fig. 4:**
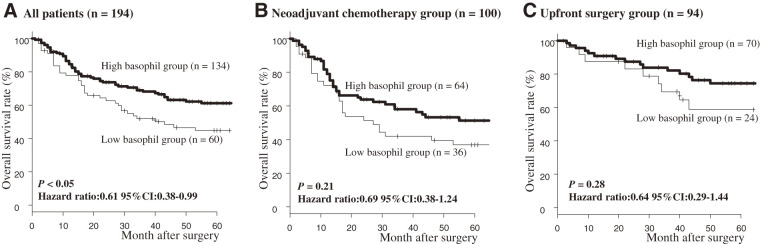
Comparison of overall survival between low- and high-basophil groups. (**A**) All patients, (**B**) neoadjuvant chemotherapy group, and (**C**) upfront surgery group. CI: confidence interval

**Fig. 5:**
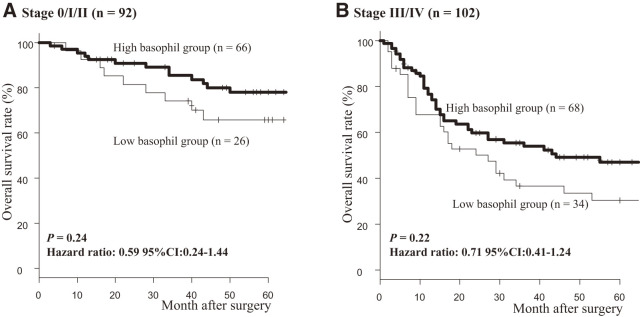
Comparison of overall survival between low- and high-basophil groups according to tumor stages. CI: confidence interval

In the overall cohort, OS was significantly worse in the low-basophil count group compared with the high-basophil count group (*P* <0.05; **Fig. 4A**). Among patients who received NAC and those who underwent upfront surgery, no statistically significant differences in OS were observed between the low- and high-basophilgroups (*P* = 0.21 and 0.28, respectively; **Figs. 4B** and **4C**). However, a trend toward poorer prognosis was noted in the low-basophil group. When patients were grouped by clinical stage (0/I/II vs stage III/IV) and OS was evaluated (**Fig. 5**), no statistically significant differences emerged; however, the low-basophil group consistently demonstrated a trend toward poorer prognosis (stage 0/I/II: *P* = 0.24; stage III/IV: *P* = 0.22).

Univariate analysis of OS in the entire cohort identified deep tumor invasion, lymph node metastasis, high CRP level, and low-basophil count as significant adverse prognostic factors (**Table 2**). In multivariate analysis, deep tumor invasion (*P* <0.01), elevated CRP (*P* <0.05), and low-basophil count (*P* <0.05) remained independent predictors of poor prognosis (**Table 2**).

**Table 2:** Univariate and multivariate analysis of clinicopathological factors to predict overall survival in all patients

Variables	Univariate *P*-value^a^	Multivariate analysis	
		HR (95% CI)	*P*-value^b^
Age			
≥65/<65	0.99		
Gender			
Male/Female	0.13		
Tumor depth			
cT2T3T4/cT0T1	<0.01	3.75 (1.61–8.76)	<0.01
Nodal status			
cN1N2N3/cN0	<0.01	1.26 (0.70–2.26)	0.44
C-reactive protein			
High/Low	<0.01	1.64 (1.00–2.69)	<0.05
Basophil			
High/Low	<0.05	0.61 (0.38–0.99)	<0.05
Neutrophil			
High/Low	0.14		
Lymphocyte			
High/Low	006		
Eosinophil			
High/Low	0.55		

^a^Log-rank analysis.

^b^Cox proportional hazards regression analysis.

CI: confidence interval; HR: hazard ratio

## Discussion

In this study, the optimal cutoff value of peripheral basophil count as a prognostic factor in ESCC was determined to be 17.4/μL. This threshold is generally consistent with values reported in other malignancies (colorectal cancer: 10–15/μL; gastric cancer: 20–26/μL; esophageal cancer: 22/μL), supporting its validity.^13–17)^ Moreover, low-basophil count emerged as an independent predictor of poor prognosis in ESCC. The cutoff value for basophils was determined by ROC analysis, but the area under the receiver-operating characteristic curve (AUC) was low at 0.58. However, even if the AUC is low, if the effect on prognosis is useful in clinical practice, we believe that this research is meaningful. Subgroup analyses stratified by NAC and upfront surgery further demonstrated a consistent trend toward worse outcomes among patients with low-basophil counts in both treatment groups.

A reduction in peripheral blood basophil counts may primarily reflect mobilization of basophils into the tumor microenvironment, although impaired bone marrow function in advanced cancer could also play a role. Previous studies of hematological parameters in cancer have suggested associations between tumor aggressiveness and systemic inflammation.^19)^ CRP is a well-established biomarker of inflammation, and elevated CRP levels have been consistently associated with poor prognosis in ESCC.^6)^ In the present study, however, no significant correlation was observed between basophil counts and CRP levels, indicating that basophil counts may represent an independent prognostic factor distinct from conventional inflammatory markers. Although the low-basophil group was significantly associated with a low neutrophil count, the basophil count itself was not a significant prognostic factor for OS in either univariate or multivariate analyses. However, the basophil counts consistently emerged as an independent predictor of poor prognosis, even after adjusting for existing inflammation-related markers. These results suggest that the basophil count may reflect prognostic information different from that of conventional neutrophil-related inflammatory markers. Although subgroup analysis stratified by NAC and upfront surgery groups did not reveal statistically significant differences, a consistent trend favoring the high-basophil count group was observed across all subgroups. Considering that a low-basophil count was also an independent prognostic factor in the overall multivariate analysis, we believe that these results are clinically significant, despite limitations in the statistical power of the subgroup analysis.

Adjuvant therapies for ESCC with poor prognosis have advanced considerably in recent years.^20–23)^ In the present study, subgroup analyses of patients receiving NAC and those undergoing upfront surgery consistently demonstrated a trend toward worse prognosis in the low-basophil count group. These findings suggest limited efficacy of CF-based NAC in patients with low-basophil counts, highlighting the potential need for treatment intensification. Preoperative or postoperative strategies, including radiotherapy or immune checkpoint inhibitors (ICIs), may offer clinical benefits in this subset of patients. In this study, NAC predominantly consisted of CF therapy, and no patients received docetaxel, cisplatin, and 5-fluorouracil (DCF). Currently, however, DCF therapy has become the standard NAC regimen^24)^ and has been shown to prolong both OS and recurrence-free survival compared with CF.^25)^ Thus, DCF-based NAC may provide better outcomes in patients with low-basophil counts. Moreover, emerging evidence indicates that perioperative use of ICIs, either alone or in combination with radiotherapy or chemoradiotherapy, may enhance therapeutic efficacy.^26,27)^ Tumor-infiltrating basophils may also serve as a predictive marker for ICI responsiveness. Further studies are warranted to clarify the relationship between basophils and response to immunotherapy.

Our findings demonstrate that low-basophil counts are associated with poor prognosis in ESCC, offering potential value for risk stratification and treatment optimization in the preoperative setting. As basophil counts can be readily obtained through routine complete blood counts, without involving additional cost or procedures, they represent a minimally invasive, rapid, and practical prognostic biomarker.

Some limitations of this study should be acknowledged. First, this was a single-center, retrospective observational study, and the potential for selection bias cannot be excluded. Second, we did not comprehensively evaluate the dynamics of basophils or their interactions with the tumor immune microenvironment. Future research should include multicenter prospective studies as well as basic investigations into the role of basophils in tumor immunity.

In conclusion, preoperative low-basophil counts were identified as an independent poor prognostic factor in ESCC. Subgroup analyses of patients receiving NAC and those undergoing upfront surgery consistently showed a worse prognosis in patients with low-basophil counts. These findings suggest that basophil count may serve as a useful prognostic indicator. Future studies should aim to develop stratified treatment strategies incorporating basophil count alongside other inflammation-related biomarkers and to further assess the efficacy of DCF-based NAC and immunotherapy in this context.

## Declarations

### Data availability

The data supporting our findings are available from the corresponding author upon reasonable request.

## References

[1] Allin KH, Nordestgaard BG. Elevated C-reactive protein in the diagnosis, prognosis, and cause of cancer. Crit Rev Clin Lab Sci 2011; 48: 155–70. 22035340 10.3109/10408363.2011.599831

[2] Yamashita S, Okugawa Y, Mizuno N, et al. Inflammatory BurdenIndex as a promising new marker for predicting surgical and oncological outcomes in colorectal cancer. Ann Gastroenterol Surg 2024; 8: 826–35. 39229558 10.1002/ags3.12829 PMC11368506

[3] Shimada H, Takiguchi N, Kainuma O, et al. High preoperative neutrophil-lymphocyte ratio predicts poor survival in patients with gastric cancer. Gastric Cancer 2010; 13: 170–6. 20820986 10.1007/s10120-010-0554-3

[4] Li B, Zhou P, Liu Y, et al. Platelet-to-lymphocyte ratio in advanced cancer: review and meta-analysis. Clin Chim Acta 2018; 483: 48–56. 29678631 10.1016/j.cca.2018.04.023

[5] Fu X, Li T, Dai Y, et al. Preoperative systemic inflammation score (SIS) is superior to neutrophil to lymphocyte ratio (NLR) as a predicting indicator in patients with esophageal squamous cell carcinoma. BMC Cancer 2019; 19: 721. 31331297 10.1186/s12885-019-5940-6 PMC6647281

[6] Huang W, Wu L, Liu X, et al. Preoperative serum C-reactive protein levels and postoperative survival in patients with esophageal squamous cell carcinoma: a propensity score matching analysis. J Cardiothorac Surg 2019; 14: 167. 31533862 10.1186/s13019-019-0981-0 PMC6751901

[7] Cupp MA, Cariolou M, Tzoulaki I, et al. Neutrophil to lymphocyte ratio and cancer prognosis: an umbrella review of systematic reviews and meta-analyses of observational studies. BMC Med 2020; 18: 360. 33213430 10.1186/s12916-020-01817-1 PMC7678319

[8] Nomoto D, Baba Y, Akiyama T, et al. Adapted systemic inflammation score as a novel prognostic marker for esophageal squamous cell carcinoma patients. Ann Gastroenterol Surg 2021; 5: 669–76. 34585051 10.1002/ags3.12464 PMC8452479

[9] Siracusa MC, Kim BS, Spergel JM, et al. Basophils and allergic inflammation. J Allergy Clin Immunol 2013; 132: 789–801; quiz, 788. 24075190 10.1016/j.jaci.2013.07.046 PMC3903395

[10] Karasuyama H, Mukai K, Obata K, et al. Nonredundant roles of basophils in immunity. Annu Rev Immunol 2011; 29: 45–69. 21166539 10.1146/annurev-immunol-031210-101257

[11] Sektioglu IM, Carretero R, Bulbuc N, et al. Basophils Promote Tumor Rejection via Chemotaxis and Infiltration of CD8+ T Cells. Cancer Res 2017; 77: 291–302. 27879269 10.1158/0008-5472.CAN-16-0993

[12] Marone G, Gambardella AR, Mattei F, et al. Basophils in tumor microenvironment and surroundings. Adv Exp Med Biol 2020; 1224: 21–34. 32036602 10.1007/978-3-030-35723-8_2

[13] Maruyama S, Okamura A, Kanie Y, et al. Prognostic significance of circulating basophil counts in patients who underwent esophagectomy for esophageal cancer. Langenbecks Arch Surg 2023; 408: 235. 37329456 10.1007/s00423-023-02977-3

[14] Wu C, Qiu Y, Zhang R, et al. Association of peripheral basophils with tumor M2 macrophage infiltration and outcomes of the anti-PD-1 inhibitor plus chemo therapy combination in advanced gastric cancer. J Transl Med 2022; 20: 386. 36058929 10.1186/s12967-022-03598-y PMC9441040

[15] Kawano M, Oshima Y, Shiratori F, et al. Association of circulating basophil count with gastric cancer prognosis. J Gastrointest Cancer 2025; 56: 54. 39869243 10.1007/s12029-025-01171-6

[16] Wu J, Ge XX, Zhu W, et al. Values of applying white blood cell counts in the prognostic evaluation of resectable colorectal cancer. Mol Med Rep 2019; 19: 2330–40. 30664202 10.3892/mmr.2019.9844

[17] Gao L, Yuan C, Fu J, et al. Prognostic scoring system based on eosinophil- and basophil-related markers for predicting the prognosis of patients with stage II and stage III colorectal cancer: a retrospective cohort study. Front Oncol 2023; 13: 1182944. 37519795 10.3389/fonc.2023.1182944 PMC10375403

[18] Kanda Y. Investigation of the freely available easy-to-use software ‘EZR’ for medical statistics. Bone Marrow Transplant 2013; 48: 452–8. 23208313 10.1038/bmt.2012.244 PMC3590441

[19] Hussain SP, Harris CC. Inflammation and cancer: an ancient link with novel potentials. Int J Cancer 2007; 121: 2373–80. 17893866 10.1002/ijc.23173

[20] Watanabe M, Otake R, Kozuki R, et al. Recent progress in multidisciplinary treatment for patients with esophageal cancer. Surg Today 2020; 50: 12–20. 31535225 10.1007/s00595-019-01878-7 PMC6952324

[21] Kelly RJ, Ajani JA, Kuzdzal J, et al. Adjuvant nivolumab in resected esophageal or gastroesophageal junction cancer. N Engl J Med 2021; 384: 1191–203. 33789008 10.1056/NEJMoa2032125

[22] Tsuji T, Matsuda S, Takeuchi M, et al. Updates of perioperative multidisciplinary treatment for surgically resectable esophageal cancer. Jpn J Clin Oncol 2023; 53: 645–52. 37282626 10.1093/jjco/hyad051

[23] Okui J, Nagashima K, Matsuda S, et al. Investigating the synergistic effects of immunochemotherapy in esophageal squamous cell carcinoma. Esophagus 2025; 22: 188–97. 39966261 10.1007/s10388-025-01113-y

[24] Kato K, Machida R, Ito Y, et al. Doublet chemotherapy, triplet chemotherapy, or doublet chemotherapy combined with radiotherapy as neoadjuvant treatment for locally advanced oesophageal cancer (JCOG1109 NExT): a randomised, controlled, open-label, phase 3 trial. Lancet 2024; 404: 55–66. 38876133 10.1016/S0140-6736(24)00745-1

[25] Matsuda S, Kitagawa Y, Takemura R, et al. Real-world evaluation of the efficacy of neoadjuvant DCF Over CF in esophageal squamous cell carcinoma: propensity score-matched analysis from 85 authorized Institutes for Esophageal Cancer in Japan. Ann Surg 2023; 278: e35–42. 35837977 10.1097/SLA.0000000000005533

[26] Kadono T, Yamamoto S, Kato K. Development of perioperative immune checkpoint inhibitor therapy for locally advanced esophageal squamous cell carcinoma. Future Oncol 2024; 20: 2097–107. 38861290 10.1080/14796694.2024.2345043 PMC11497952

[27] Ghalehtaki R, Amini A, Abyaneh R. Optimizing neoadjuvant radiotherapy for locally advanced esophageal squamous cell carcinoma: a comprehensive review on the role of concomitant or sequential immune checkpoint inhibitors. Esophagus 2025; 22: 5–18. 39562407 10.1007/s10388-024-01097-1

